# The diverse roles of C-type lectin-like receptors in immunity

**DOI:** 10.3389/fimmu.2023.1126043

**Published:** 2023-02-27

**Authors:** Michal Scur, Brendon D. Parsons, Sayanti Dey, Andrew P. Makrigiannis

**Affiliations:** Department of Microbiology and Immunology, Dalhousie University, Halifax, NS, Canada

**Keywords:** C-type lectin-like receptors, pattern recognition receptors, CLEC, NKRP1 receptors, immunity

## Abstract

Our understanding of the C-type lectin-like receptors (CTLRs) and their functions in immunity have continued to expand from their initial roles in pathogen recognition. There are now clear examples of CTLRs acting as scavenger receptors, sensors of cell death and cell transformation, and regulators of immune responses and homeostasis. This range of function reflects an extensive diversity in the expression and signaling activity between individual CTLR members of otherwise highly conserved families. Adding to this diversity is the constant discovery of new receptor binding capabilities and receptor-ligand interactions, distinct cellular expression profiles, and receptor structures and signaling mechanisms which have expanded the defining roles of CTLRs in immunity. The natural killer cell receptors exemplify this functional diversity with growing evidence of their activity in other immune populations and tissues. Here, we broadly review select families of CTLRs encoded in the natural killer cell gene complex (NKC) highlighting key receptors that demonstrate the complex multifunctional capabilities of these proteins. We focus on recent evidence from research on the NKRP1 family of CTLRs and their interaction with the related C-type lectin (CLEC) ligands which together exhibit essential immune functions beyond their defined activity in natural killer (NK) cells. The ever-expanding evidence for the requirement of CTLR in numerous biological processes emphasizes the need to better understand the functional potential of these receptor families in immune defense and pathological conditions.

## Introduction

Immune surveillance by cells of the innate immune system is guided by an extensive repertoire of highly conserved pattern recognition receptors (PRRs). These PRRs can recognize a diverse variety of ligands composed of carbohydrates, lipids, proteins, and glycoproteins. The PRRs are well-established as receptors of pathogen-associated molecular patterns (PAMPs), or damage-associated molecular patterns (DAMPs) from dead and dying cells (1, 2). Most PRRs have been categorized based on their domain architecture, phylogeny, function, or cellular location. Among these categories are five major families: the Toll-like receptors (TLRs), C-type lectin (CLEC) receptors (the CLRs and the related C-type lectin-like receptors CTLRs), retinoic acid-inducible gene 1-like receptors (RLRs), nucleotide-binding oligomerization domain leucine-rich repeat receptors (NLRs) (3), and absent in melanoma (AIM)-like receptors (ALRs), which have been widely accepted as another class of PRRs. These receptor families are evolutionarily well-conserved, yet display distinct and overlapping interactions with different ligands, producing a cellular early detection system with a repertoire of sensors that is large, varied, and usually accompanied by multiple redundancies. Nowhere is this more typified than in the CLEC receptors, which demonstrate remarkable diversity in ligand recognition and signaling functions that elicit responses beyond simple immune activation.

The superfamily of CLEC receptors contains numerous groups of the most evolutionarily diverse PRRs in the mammalian repertoire (4). There have been several attempts to group this highly diverse receptor family into more manageable divisions. The most recent iteration resulted in the creation of 17 different sub-categories based on phylogeny and structural similarities (5). A defining characteristic of CLEC receptors is the presence of a C-type lectin-like domain (CTLD) and a requirement for a Ca^2+^ ion for efficient glycan binding. However, many receptors of the C-type lectin family possess a CTLD but lack any type of ionic requirement for ligand recognition and are therefore referred to as C-type lectin-like receptors (CTLRs) (6). Despite this distinction, there currently exists significant overlap as to whether a receptor is a canonical calcium-dependent CLEC or a CTLR as many of these receptors’ binding domain structures have not been fully elucidated (7). Receptors previously classified as CLECs or CTLRs continue to be reshuffled as more information is determined and this is shown in the literature where the same receptor may appear classified as both a CLEC and a CTLR depending on the time of publishing. Due to the calcium independence of CTLRs, they can recognize a large variety of ligands, including lipids and proteins, on top of the canonical carbohydrate ligands *via* their unique carbohydrate-binding domains (CBD), which constitute the vast majority of CLEC interactions (8). The canonical, but not exclusive, role for both CLECs and CTLRs is to serve as PRRs on myeloid-derived immune cells to recognize various PAMPs and initiate appropriate signaling cascades which can lead to immune activation (9). Many of these receptors are classified as collectins and selectins but some fall outside of a specific classification scheme due either to the unique role they perform or their unique, cell-specific expression pattern.

Owing to the family’s large repertoire, covering them all is beyond the scope of this review, but two groups, in particular, have been demonstrated to play an outsized role in the immune response: Group II or the Type II receptors, and the large variety of natural killer (NK) cell-associated receptors which have been categorized as the Group V class of C-type lectin receptors. The Type II receptors contain some of the earliest and most well-understood C-type lectin receptors involved in PAMP and DAMP ligand recognition, but members of which more recently have been found to recognize a more diverse pallet of ligands, mediate non-canonical and divergent signaling processes, and drive immunomodulatory processes. The NK-associated receptors within Group V contain the defining CTLD but also exhibit broadly unique characteristics that open the door to interesting protein-protein interactions and signaling possibilities. Together these groups of CLECs and CTLRs demonstrate the increasingly recognized capacity of C-type lectin in immunity.

## Diversity in ligand binding among receptors of the dectin-1 cluster

**Figure 1:**
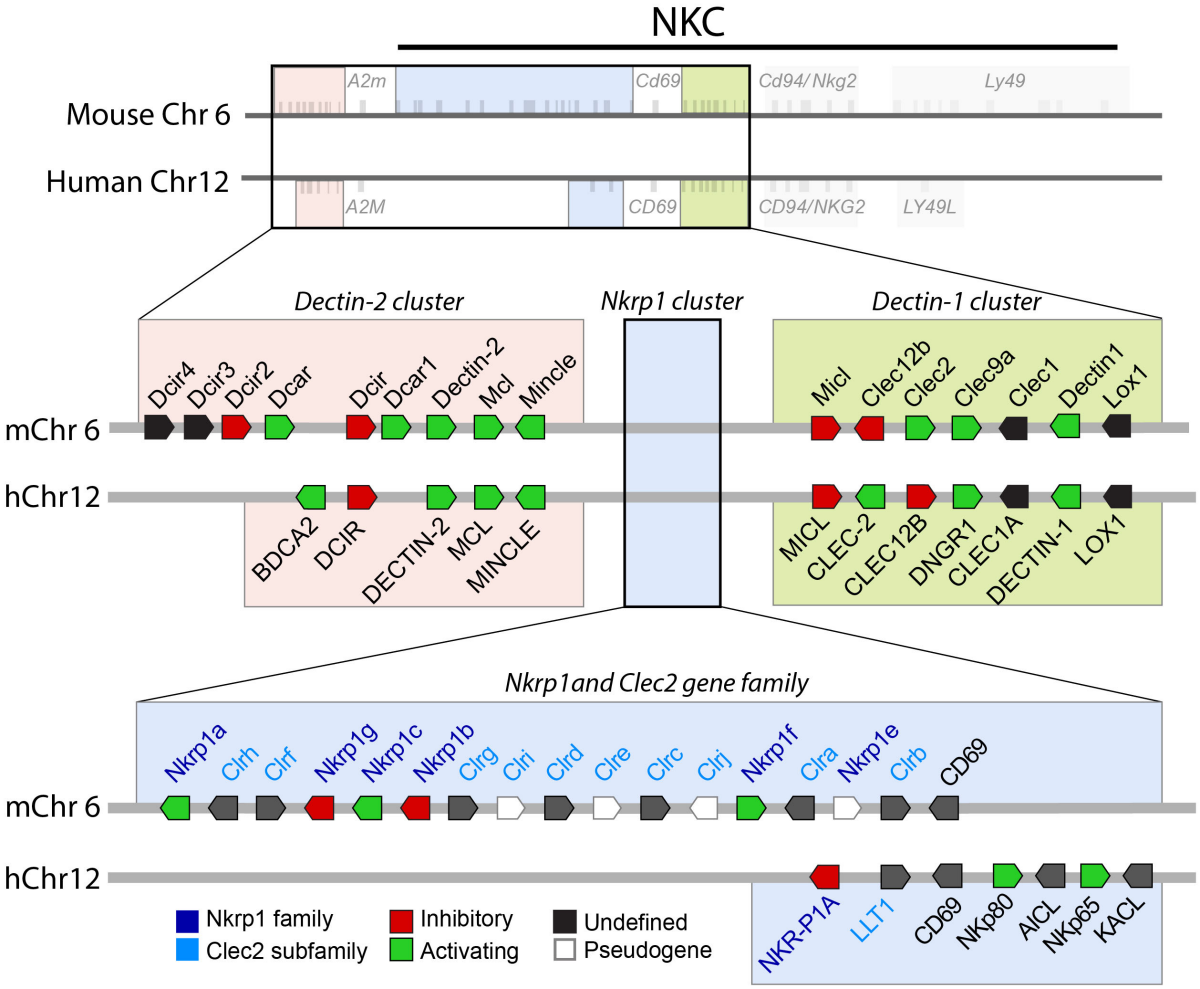
Organization of the Dectin-1, Dectin -2, and NKRP1 gene clusters. Each gene cluster is shown relative to the NKC of mouse chromosome 6 and human chromosome 12. The gene orientation, relative organization, and the activating or inhibitory function of CTLR and CLEC proteins are shown. Gene size, exact location, and relative distance are not depicted. Common gene names or gene product names are indicated.

Many of the CTLR family of receptors encoded in the NKC are functionally calcium-independent and comprise a wide array of signaling-capable molecules with a large variety of ligand specificities. The multitude of downstream responses mediated by these receptors is largely dictated by the type of ligand-bound and cell-type expressing the receptor. Among the first signaling CTLRs identified to be expressed on myeloid cells are encoded in the Dectin-1 cluster. The Dectin-1 cluster of genes is located within the mouse and human NKC between the NKRP1 receptor family and the NKG2 receptors and encodes seven CTLRs (**Figure 1**). These receptors include MICL (CLEC12A), CLEC-2, CLEC-12B, DNGR1 (CLEC9A), CLEC1 (MelLec), DECTIN-1 (CLEC7A), and LOX-1 (OLR1) and are present in both humans and mice. While the receptors of the Dectin-1 and Dectin-2 clusters have been found to play a role in a diverse range of immune functions, much of their functional diversity can be attributed to the broad range of ligands they can engage in addition to the growing list of canonical and non-canonical interactions they have with PAMPs and DAMPs.

One such example includes the well-characterized DECTIN-1 receptor, which is expressed on myeloid cells and B-cells across higher-order mammals and rodents. Notably, DECTIN-1 exemplifies a canonical CTLR because it contains a single CBD, which is identical to the prototypical calcium-dependent receptor, DC-SIGN, but unlink DC-SIGN, was not found to contain a Ca^2+^ ion binding domain and therefore is more similar to the NK-cell lectin receptors and other MHC-I binding proteins (10). DECTIN-1 is now understood to recognize a variety of ligands but was initially defined as a key immune receptor in the recognition of β-glucans to mediate anti-fungal immunity (11). The expansion of DECTIN-1 ligands beyond β-glucans revealed the role of these receptors in numerous pathologies, such as cancer, metabolic disorders, and autoimmunity. Among the non-canonical ligands reported to be recognized by DECTIN-1 are DAMPs such as Annexins (12), the filamentous protein Tropomyosin (13), and tumor-associated N-glycans (14). Contrary to the typical DAMP recognition outcome of proinflammatory signaling, the binding of apoptotic cells by DECTIN-1 through recognition of annexins 1, 5, and 13, or the presence of tropomyosin in an allergy model in mice on drives immunosuppressive and immunotolerogenic outcomes.

The Dectin-1 cluster member MICL, or the myeloid inhibitory C-type lectin-like protein, is a CTLR that is expressed primarily on monocytes, granulocytes, and NK cells. It exhibits a wide mammalian distribution as well as orthologs identified in birds and reptiles (15). Most CTLRs have some role in binding pathogen-derived molecules; however, MICL exhibits extensive binding activity for endogenous ligands (16). This ligand-binding specificity is characteristic of receptors with potential immunomodulatory roles. Indeed, a later report demonstrated that MICL plays a role in inhibiting innate immune responses, and in particular, a pivotal role in regulating runaway inflammation during rheumatoid arthritis (17). Similarly, (18) showed that MICL recognition of uric acid crystals, characteristic DAMPs of dying cells, limited inflammatory outcomes. Aside from its binding of endogenous ligands, MICL also engages PAMPs. For example, the inhibitory response through MICL has been shown to provide an advantage to Mycobacterium, as MICL recognition of the mycobacterial cell wall mycolic acids resulted in a dampened immune response that permitted the establishment of chronic mycobacterial infection (19). Also, (20) showed that MICL can bind the malarial PAMP hemozoin, in a manner that drives a CD8^+^ T cell response to experimental cerebral malaria. The fact that this receptor plays such a prominent immunomodulatory role instead of the traditionally associated pro-inflammatory role of CTLR highlights the diversity not only of CLEC and CTLR-ligand interactions but also of vastly different and complex roles CTLRs play in immune homeostasis.

The CTLR, CLEC-2, is conserved across higher-order mammals and is mainly expressed on platelets and dendritic cells (DCs). For many years the only known ligand of CLEC-2 was podoplanin, whose binding serves to activate platelets and promote aggregation (21). However, recently DECTIN-1 was identified as a ligand of CLEC-2 (22). Specifically, CLEC-2 was observed to interact with an *O-*glycosylated moiety on DECTIN-1. This type of heterophilic binding between CLEC and CTLR receptors resembles the self-recognition interactions mediated by NK cell-associated receptors such as with the NKRP1 and Clr family. On DCs, CLEC-2 appears to work synergistically with LPS sensor TLR-4 to enhance the production of IL-10, suggesting a role in inflammation resolution towards the end stage of gram-negative bacterial infection (23). On top of its direct immune function, CLEC-2 has been implicated in the development and maintenance of bone marrow hematopoietic stem cells (24). The authors were able to positively link CLEC-2 expression on megakaryocytes to the secretion of thrombopoietin, thus providing an explanation for the gradual decline in hematopoietic stem cells observed in CLEC-2 deficient mice (24).

Among the prototypical CTLRs, the LOX-1 receptor, which is found in most mammals and exhibits expression on macrophages and endothelial cells, is unique in the type of ligand it recognizes. LOX-1 serves mainly as a low-affinity receptor for oxidized-low-density lipoprotein (ox-LDL) and is implicated in the formation of atherosclerotic lesions (25). Notably, this is similarly reflected in the alternate binding activity of other PRRs, such as TLR-4, which can induce the transition of macrophages into foam cells in an ox-LDL-mediated manner, contributing to atherosclerotic plaque formation (26). Likewise, other studies have outlined the importance of the TLR-2, TLR-4, and CD36 axis in macrophage dysregulation leading to foam cell formation achieved by the binding of lipid species not normally associated with TLRs (26–28). Although macrophages typically express high levels of the LOX-1 receptors, DCs also express LOX-1, albeit at lower levels. However, in this context, LOX-1 demonstrates the ability to bind Hsp70 and promote antigenic cross-presentation on DCs (29), thus making LOX-1 a potential target molecule for cancer therapies that rely on immunogenic cell death to spur anti-tumor responses.

Aside from the aforementioned members of the Dectin-1 cluster, there are few to no identified ligands for the CTLRs DNGR1, CLEC12B, and CLEC-1. To date, F-actin is the only known ligand of DNGR1 (30) and no known ligands to CLEC12B and CLEC-1 have been defined. As a ligand for DNGR1, F-actin represents a characteristic DAMP of dying necrotic cells. Notably, DNGR1 engagement with F-actin does not drive proinflammatory outcomes. As with the non-canonical ligand binding described in the highly related CTLR Dectin-1, DNGR1 binding drives processes that limit inflammation, possibly as a means of regulation of heterologous inflammatory signaling pathways (31). Although a ligand for CLEC12B has not yet been defined, the endogenous ligand caveolin-1 is thought to be recognized by CLEC12B (32). CLEC-1 has confounded researchers for quite some time as it still has no known ligand and its signaling remains poorly characterized despite being discovered more than a decade ago. CLEC-1 appears to be expressed on most myeloid cells and its expression is downregulated by inflammatory stimuli and upregulated by resolving cytokines, specifically TGF-β (33). This receptor is also one of the few that is predominantly found intracellularly, adding further confusion as to its function and its ligand (34). CLEC-1 receptor deficiency experiments showed an upregulation in IL-12 secretion by DCs contributing to further exacerbation of Th1 responses, which suggests that CLEC-1 functions as an inhibitory receptor in DCs and whose function is to limit Th responses (35, 36).

## Diversity in ligand binding among receptors of the dectin-2 cluster

Encoded on the opposite side of the NKRP1 gene family from the Dectin-1 cluster are the genes belonging to the Dectin-2 cluster (**Figure 1**). While the receptors of the Dectin-1 cluster are widely expressed on immune and non-immune cells alike, the receptors of the Dectin-2 cluster are exclusively expressed on immune cells. The Dectin-2 cluster encodes genes of five human and nine mouse receptors (respectively, the BDCA2, DCIR, DECTIN-2, MCL, and MINCLE receptors in humans and Dcir4, Dcir3, Dcir2, Dcar, Dcir, Dcar1, Dectin-2, Mcl, and Mincle in mice).

Notably, all but one of the human Dectin-2 family of receptors mediate signal transduction indirectly *via* activating motif-containing adaptors. One of these activating receptors, MINCLE (Clec4e), which is expressed on mammalian myeloid cells and possibly B-cells, is a prototypical example of a CLR with non-canonical binding attributes. As a characteristic PRR, MINCLE was originally described to mediate immunity to the fungi *Candida albicans* (37) and was found to directly recognize PAMPs of the cell wall of *Mycobacterium tuberculosis*, such as glycolipid trehalose-6,6′-dimycolate (38). More recent evidence has shown that MINCLE can bind a wide variety of other endogenous ligands including DAMPs as products of cell death such as cholesterol sulfate (39), β-glycosylceramides (40), cholesterol crystals (41), and SAP130 (42, 43). MINCLE exhibits a particularly interesting signaling ability, as it canonically associates with the ITAM-bearing FcRγ to signal but can also form heterodimers with MCL (Clec4d) to promote efficient phagocytosis and synergistically increase ligand affinity of both receptors (44). The consequences of signal transduction through MCL and MINCLE typically drive inflammation through the production of proinflammatory cytokines and chemokines.

The DECTIN-2 receptor is one of the most well-characterized members of the Dectin-2 cluster. Expressed on macrophages, DCs, neutrophils, monocytes, and other myeloid cells, DECTIN-2 exhibits characteristics of lectin calcium-dependent ligand binding through its extracellular CBD. While relatively few ligands are described for DECTIN-2, it shows a high affinity for microbial and fungal PAMPs, such as α-1,2 and α-1,4 mannose, (45), N- and O-linked glycans, and other glycoproteins (46) and elicits characteristic activating responses to drive protective cellular immune responses to pulmonary infections. Beyond these PAMPs, DECTIN-2 is speculated to also have endogenous ligands possibly on regulator T cell subsets, whereby its expression on Langerhans cells plays a role in immune suppression of skin contact hypersensitivity responses (47).

The dendritic cell immunoreceptor, DCIR (Clec4a2), is predominantly expressed on DCs and osteoclasts. As DCIR contains a canonical extracellular CBD, it carries the potential to recognize glycan structures in a calcium-dependent manner. Indeed, DCIR has shown a binding affinity for glycan expressed on CHO cells (48). Functionally, DCIR demonstrates inhibitory signaling roles and was originally defined to regulate immune responses generated through TLRs (49). Recently DCIR was found to recognize biantennary asialo-N-glycans present on myeloid and bone cells (50). The binding of this endogenous glycan by DCIR results in the suppression of DC and osteoclast differentiation, demonstrating a role for this receptor in immune development as well as immune regulation.

BDCA2 presents another puzzling case as it is present on human plasmacytoid DCs (pDCs) but lacks a mouse homolog (51). The canonical ligands for BDCA2 include CpG DNA, similar to TLR-9, as well as galactose residues which happen to be preferentially expressed on many tumor cells (51, 52). However, a study has demonstrated that BDCA2 can bind glycan residues present on many immunoglobulin species, thus acting as an Fc receptor and a sink for antibodies in response to excess Ig presence in the bloodstream due to runaway inflammation. (53). These examples from the Dectin clusters of receptors reveal the flexible and highly complex nature of both CLEC and CTLRs. The ability to bind ligands ranging from lipids, proteins, and glycoproteins plus the ability to form heterodimers provide these receptor families with a wide variety of cell type-dependent effects, which also translates into the variable nature of CLEC and CTLR signaling.

## Canonical CLEC and CTLR signaling

**Figure 2:**
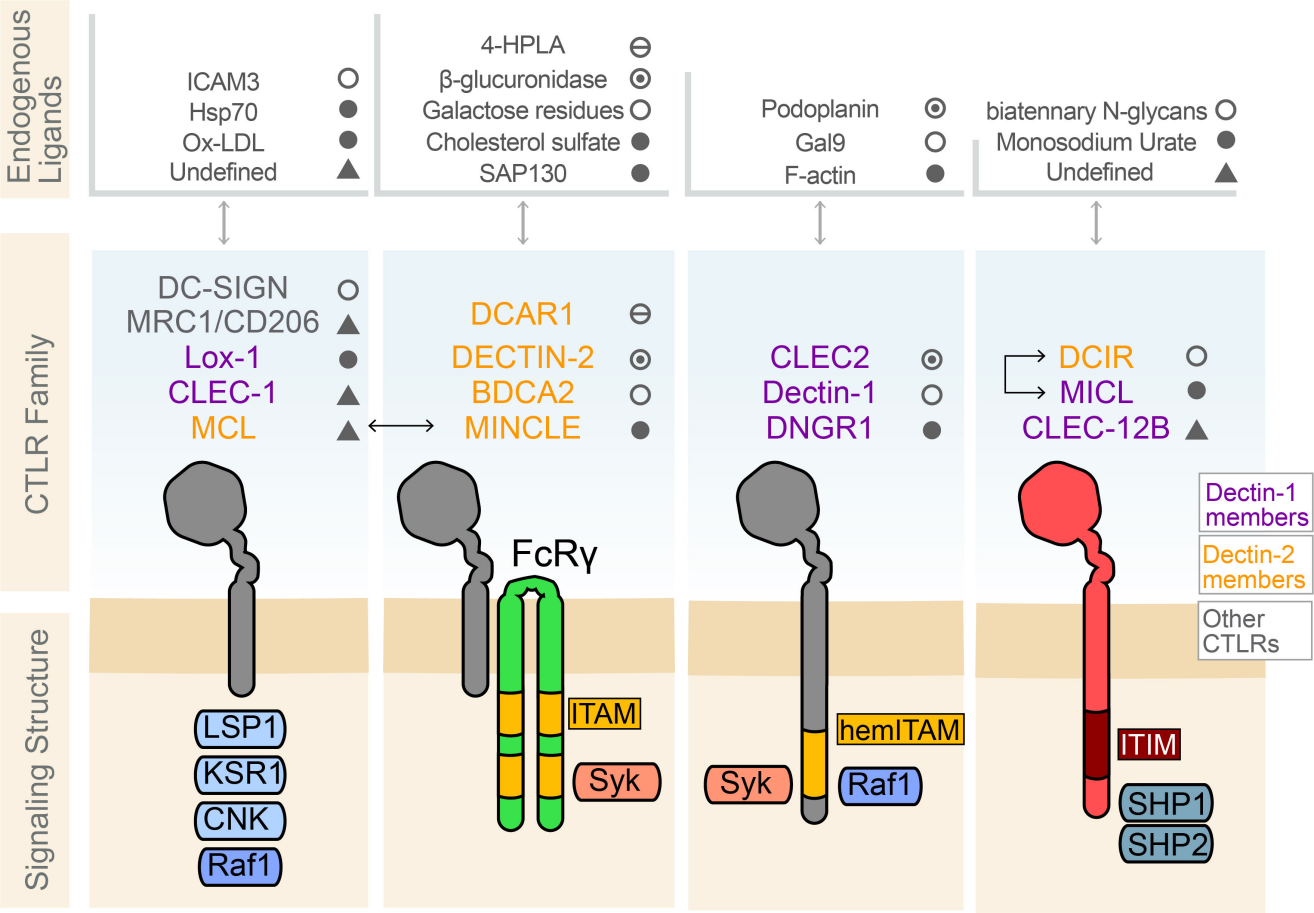
Select C-type lectin-like receptors, their known endogenous ligands, and their signaling structure. Several common CTLRs protein signaling structures are shared across the C-type lectins, including those within the Dectin-1 and Dectin-2 clusters. These include the CTLRs in which recognizable cytoplasmic signaling motifs are absent, such as DC-SIGN, Mannose Receptors (MRC1), LOX-1, CLEC-1, and MCL. The CTLRs DCAR1, DECTIN-2, BDCA2 in humans, and MINCLE, dimerize with ITAM-containing FcRg to initiate Syk kinase recruitment to trigger downstream signaling cascades. MCL and MINCLE, dimerize with FcRγ to take advantage of the ITAM present on the FcRγ receptor for signaling. CTLRs such as CLEC2, Dectin-1, and DNGR1 contain a hemITAM to signal. Dectin-1 contains domains for signaling through Raf-1 to mediated signaling involved in phagocytosis. MICL, DCIR, and CLEC-12B bear ITIM domains on their cytoplasmic tails which are known to recruit src-homology containing phosphatase 1/2 (SHP1/2). These phosphatases then play a role in signal modulation by dephosphorylating Syk or the further downstream activating factor NF-κB. MICL and DCIR heterodimerization have been shown to mediate this inhibitory signaling. Examples of the endogenous ligands known for some of these CTLRs are specified.

Prototypical immune activation signaling is propagated by receptors that signal through associated adaptors that contain immunotyrosine activating motifs (ITAM). Several activating CTLRs also exhibit this mechanism of signaling through ITAM-containing adaptors. These ITAMs contain the consensus amino acid sequence YxxL/I (X_6-8_)YxxL/I (x denotes any amino acid) (54). CTLRs that drive inhibitory signaling contain cytoplasmic domains with immunotyrosine inhibitory motifs (ITIM), a motif with the consensus sequence (I/V/L/S)xYxx (L/V) (55). Inhibitory CTLRs are in the minority and therefore have been less studied, as evidenced by the lack of defined ligands for the most characteristic inhibitory CTLRs, DCIR and MICL (**Figure 2**).

Dimerization of signaling ITAM- or ITIM-bearing receptors is typically needed to achieve the phosphorylation of the docking sites for signaling adaptors (56). The recruitment of adaptor proteins to CLECs and CTLRs receptors following ligand binding and dimerization initiates a relay of downstream signaling cascades. The vast majority of canonical activating CLEC signaling takes place *via* the recruitment of the non-receptor tyrosine kinase Syk to the ITAM, with the ITAM either incorporated into the receptor itself or contained within an adaptor protein such as FcRγ. This membrane proximal signal propagation can produce a wide variety of downstream activating functions depending on the ligand and cell type expressing the receptor (57). This is in contrast to ITIM-bearing CTLRs which tend to recruit the SH2 domain-containing phosphatases, SHP-1 and SHP-2, or the SH2 domain-containing inositol 5’ phosphatase, SHIP-1, to their ITIM domains, which produces inhibitory signaling that suppresses activation or cellular responses (58).

## Non-canonical CLEC and CTLR signaling

Outside of these canonical signaling pathways, CLECs and CTLRs display some unusual signal transduction mechanisms that permit variability in their signaling and flexibility in their downstream functions. DC-SIGN and Dectin-1 signaling offer examples of the flexibility and wide variety of potential signaling mechanisms present in this receptor family class. DC-SIGN in particular offers a very intriguing case study as to the flexibility of CLEC/CTLR signaling since it does not possess any known signaling motifs; relying exclusively on a cascade of scaffolds and mediators to produce downstream effects. If DC-SIGN binds mannosylated ligands it will associate with the LSP1-KSR1-CNK signaling complex leading to Raf-1 activation and cellular activation and enhanced pro-inflammatory responses (59). In contrast, the binding of fucosylated ligands causes the signal complex to fall apart leaving only LSP1 in association with DC-SIGN. This complex recruits IKKϵ which causes repression of Bcl-mediated TLR signaling and promotes secretion of anti-inflammatory cytokines and overall Th2 skewing of cell activation (60). Another non-canonical signaling mechanism is exhibited by DECTIN-1, which can preferentially recruit Raf-1 instead of Syk to order to drive non-canonical NF-κB signaling in helper T-cells (61), further demonstrating the flexibility of a CTLR to drive two distinct activating signaling processes from a single receptor.

CLEC and CTLR signaling mechanics have been an active area of research that has shed light on the flexibility of these receptors to produce signaling events that appear to deviate from the norm and are in some cases even paradoxical. CLEC/CTLR signaling is influenced by not only the ligand but also whether homodimerization or heterodimerization occurs, which can have significant implications for downstream signaling by potentially turning an ITAM-bearing receptor responsible for an activating cascade into an inhibitory cascade. Such is the case of MICL (CLEC12A), where engagement with TLR-2 causes an inflammatory cascade (16) while engagement with CD40 causes an inhibitory cascade within DCs (62). In addition to expanding the functional capabilities of each receptor, heterodimerization can also change the ligand recognition capacity of individual CTLRs from that observed by receptor homodimerization. For example, the Dectin-2 and Dectin-3 homodimers have a well-defined affinity for β-glucans present in yeast cell walls (57). A recent report demonstrated that the Dectin-2,3 heterodimer configuration was much more capable of recognizing *C. albicans* fungal infection than either homodimer alone, with the heterodimer also able to elicit a stronger NF-kB-mediated immune response (63).

Another example of non-canonical signaling among the CTLRs is the notion of activating signaling by ITIM-bearing receptors. Once again, MICL, normally a suppressive receptor that controls sterile inflammation, has been found to enhance interferon responses to viral infection downstream of RIG-I (64). These opposing functions were demonstrated to be the result of a bifurcation signaling event since the signaling effect was independent of SHP1/2 activation indicating the need for a different downstream kinase or phosphatase activation (64). The activating function of ITIM-bearing CLTRs has also been demonstrated through mechanisms of signaling adaptor interference. The ITIM-containing CLEC4A receptor has been shown to induce type-I interferon secretion from DC cells upon binding of specific tuberculosis-related ligands. In this context, CLEC4A may be acting as a molecular sink, in a manner that forces SHP-1/2 to dock thereby not allowing them to exert downstream inhibitory functions (65). Lastly, the Ly49Q receptor, another ITIM-bearing receptor, that is usually associated with natural killer cell inhibition, was found to paradoxically induce IFNα secretion by pDCs (66, 67). The mechanism for this non-canonical signaling is currently unknown but is thought that perhaps Ly49Q may act in an activating manner in the presence of TLR-7 and TLR-9 agonists or by working in conjunction with the DAP-12 activating adaptor protein (67).

This divergence from the typical signaling features is further exemplified by a CLEC signaling phenomenon referred to as non-activating hemITAM signaling, so called as it does not require receptor dimerization and allows for the binding of only a single phospho-kinase protein. This is best exemplified through the function of DNGR-1 (or CLEC9A) (68), whose hemITAM has been proposed to potentiate cross-presentation by CD11^+^ DCs to T-cells (69) as well as priming CD8^+^ T-cell memory responses (70) but is not involved in any classically defined inflammatory cascade that CLECs and CTLRs are known for (NFAT or NFκB activation). Other reports have shown that hemITAM, Syk-mediated signaling of Dectin-1 in response to fungal pathogens can be modified through the specific cellular localization of Dectin-1 isoforms (71). This represents another avenue for control of CLEC signaling depending on the location of the particular isoform of the receptor.

In contrast to the phenomenon of activating signaling from ITIM-bearing CTLRs, there are now reports of ITAM-bearing CTLRs that paradoxically signal in an inhibitory manner. An example of this is observed with the CTLR, MINCLE, which usually participates in activating immune signaling cascades through Syk upon stimulation. However, under certain conditions of low-affinity binding to *Leishmania* antigens, MINCLE can assume an inhibitory ITAM conformation which allows for transient Syk binding that results in SHP-1 recruitment and a dampening of what would normally be an activating immune response (72). A similar SHP-1-dependent response has been observed during Mannose Receptor (CD206)-mediated *M. tuberculosis* binding-induced phagocytosis. The binding of *M. tuberculosis* by the Mannose Receptor coupled to FcRγ was shown to prompt SHP-1 recruitment to the phagosome where it inhibited the degradation of the bacterium thereby allowing it to proliferate inside the macrophage (73). Taken together, the signaling diversity among the C-type lectin-like family of receptors illustrates how these receptors have evolved to incorporate significantly varied functionality. The ability of these receptors to promiscuously bind different ligands, form non-canonical hetero or homodimers, and induce contradictory signaling such as activating cascades through ITIMs or inhibiting cascades through ITAMs, makes this family of receptors an important and particularly difficult area to investigate.

## CLEC and CTLR family in NK cells

A very closely related family of CLEC receptors has been defined for their function in NK cells. Unlike T-cells, NK cells do not undergo receptor recombination and instead have all of their receptors ‘hard-coded’ into the genome within the NKC (74). The lack of recombination capacity among NK cell receptors is partly compensated by the sheer size of the NK cell receptor repertoire, which comprises several different families of related receptors that all contribute to NK-cell function. NK cells operate on a balance of input signals received from their various surface receptors as they interact with a target cell. The net summary of these integrated interactions determines whether an NK cell will engage a target cell or whether it will move on to find a different target (75). In mice, these receptors are classified into the NKRP1, NKG2, and Ly49 families of receptors (76, 77). Many of these receptors, including the NKRP1 and Clr family, fall into the C-type lectin-like receptor class, which along with the canonical CLECs discussed previously form a large and diverse family of receptors present mostly on immune cells and that carry out a wide array of immunological functions. Some of the more notable receptors in the NK family of CLECs include the Ly49 family of receptors in mice, the functional homologs of the well-known KIR family of proteins in humans (78, 79). The Ly49 family of receptors performs a similar immunosurveillance function to KIRs (80), but also has a role to play in NK cell education (81) and NK cell memory formation (82). The NKG2 family of receptors recognizes ligands associated with cell stress and other proteins expressed on the surface of target cells which may be undergoing cancerous transformation or an active viral infection. The majority of the NKG2 receptors tend to be activating, such as NKG2C and NKG2D (79). NKG2D, for example, interacts with ligands like MIC-A and MIC-B, which are often upregulated on cancer cells and virally infected cells (83). NKG2A is an example of an inhibitory NKG2 family receptor, and it tends to bind to HLA-E in humans and Qa-1*^b^* in mice in what is thought to be analogous to other NK-cell inhibitory interactions (84).

## NKRP1 and Clr receptor/ligand family

The last major NK-cell receptor family is the NKRP1 family of receptors and ligands. Also located in the NKC along with the other NK-cell receptors but the NKRP1 receptors are inherited together with their ligands on one single locus (**Figure 1**) (77). Conservation of this family is evidenced in its presence among mice, rats, dogs, cattle, and humans, indicating that this gene family plays a significant role in immune function (78). The NKRP1 receptors tend to form homodimers and are classified officially as class II transmembrane C-type lectin-like receptors (85, 86). Additionally, while the receptors are considered to be C-type lectin-like receptors their ligands (the Clr proteins) are C-type lectin-related proteins (87) which opens up interesting binding possibilities between CTLRs as well as broad functional potential. Among rodents, the locus comprises genes encoding the NKRP1 receptors Nkrp1a, g, c, b/d, and f, and the pseudogene Nkrp1e. The Clr ligands of these receptors are encoded by genes interspersed among the NKRP1 family of genes. The Clr protein family in mice consists of seven members Clr-h, -f, -g, -d, -c, -a, and -b, with Clr-i, -e, and -j defined as pseudogenes that have now lost functionality. The Clr proteins exhibit distinct and overlapping tissue-specific patterns of expression. In humans, the NKRP1 receptor family is represented by a single receptor, NKR-P1A (CD161), and its ligand the lectin-like transcript 1 (LLT1), encoded by the *CLEC2D* gene, the only functional and structural ortholog of the mouse Clr proteins. LLT1 is expressed on lymphocytes, DCs, osteoblasts, and cells of the gallbladder, digestive tract, lung, and kidney tubular epithelium.

Structurally, these molecules have been shown to interact with other proteins (77) but can also recognize high-weight carbohydrates in a calcium-independent manner (88). The NKRP1 receptors are thought primarily to form homodimers, as has been demonstrated with Nkrp1b and Clr-b, however, heterodimeric structures are theoretically possible as well (89). The human NKR-P1A and DNGR-1 appear to be the most structurally related receptors to the mouse Nkrp1b receptor (90). Significantly less is known about the mouse Clr and human LLT1 proteins in terms of structure and composition with some evidence pointing to a similarity between LLT1 and CD69 (91). However, recent structural analyses of Nkrp1b bound to Clr-b determined that optimal binding affinity is achieved through an Nkrp1b dimer interacting with two Clr-b homodimers (92). This finding has been mirrored in a recent structural analysis of the human NKR-P1A: LLT1 complexes, which provided evidence for the assembly of NKR-P1A homodimers in a formation that bridges two LLT1 proteins. However, this study also revealed a unique higher avidity binding organization by NKR-P1A that multimerizes LLT1 in a manner that can bolster effective inhibitory signaling by an otherwise weak ligand-receptor affinity interaction (93). These crystal structures provide additional evidence of the similarity and conservation between the mouse Nkrp1b and human NKR-P1A as well as the similarity between the mouse Clr-b and Clr-g proteins (92), which could imply the possibility of potential alternative binding partners for Nkrp1b.

## NKRP1 cell signaling and function

**Figure 3:**
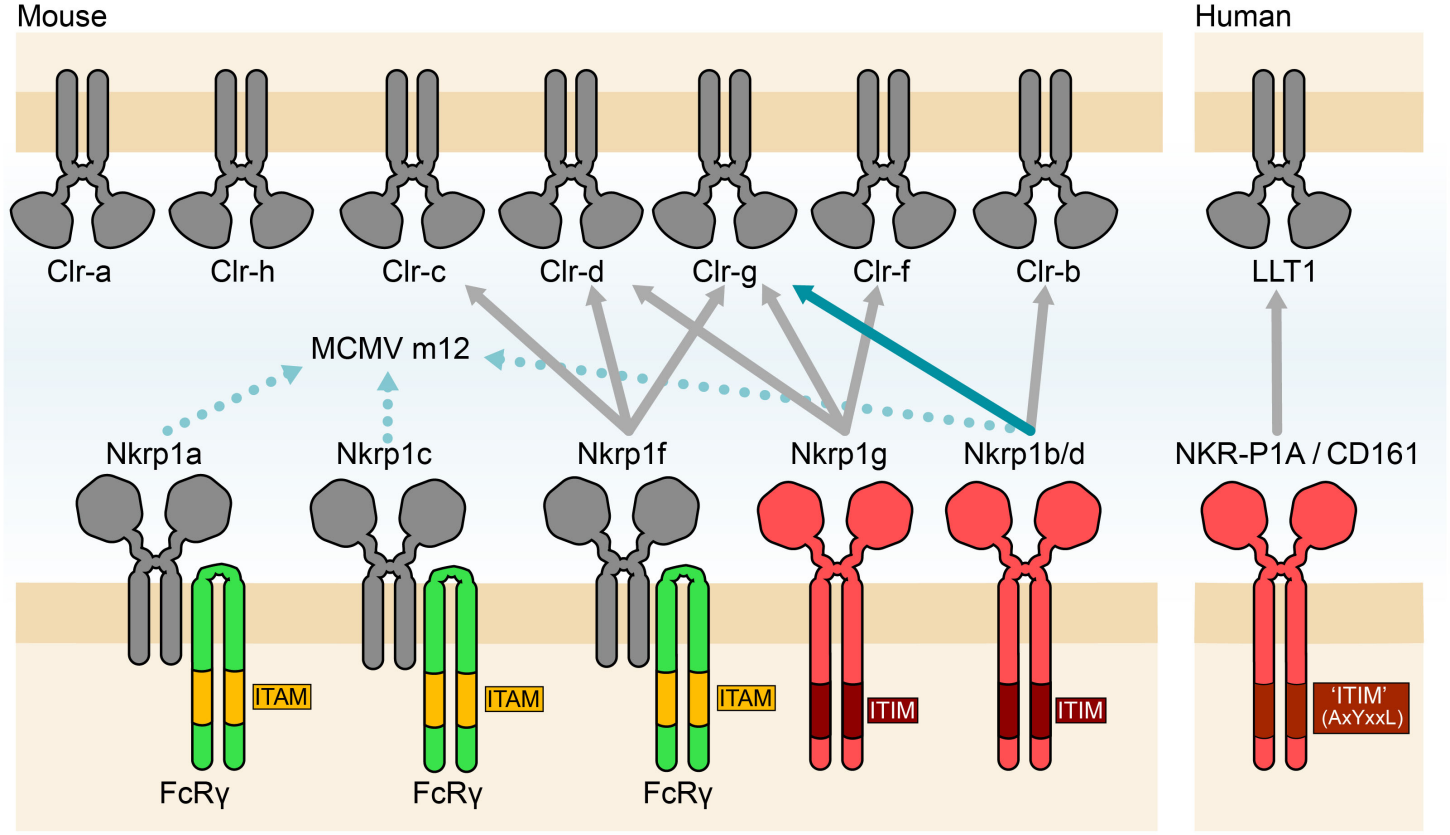
Known and posited interactions of the NKRP1 receptors and Clr/CLEC proteins. The Nkrp1f and Nkrp1g receptors have several interacting partners (Clr-c/d/g and Clr-d/g/f respectively), while Nkrp1b/d has a demonstrated interaction with Clr-b. A new interaction between Nkrp1b/d receptor expressing alveolar macrophages with Clr-g expressing lung pneumocytes was recently posited. Whereas Nkrp1a and Nkrp1c have no described endogenous ligand but are described to interact along with Nkrp1b/d, with the MCMV protein m12. The human NKR-P1A/CD161 receptor is shown with its reported ligand LLT1. All known activating NKRP1 members signal through dimerization with FcRγ to exert their activating effects, whereas the inhibitory Nkrp1g and Nkrp1b/d contain integrated ITIM domains in their cytoplasmic tail and likely exert their inhibitory effects through recruitment of SHP-1.

Similar to the Ly49 and NKG2D receptors, the NKRP1 family contains receptors that elicit either inhibitory or activating effects in NK cells. The structural similarities between the NKRP1 receptors as well as their Clr ligands suggest that there is significant potential for cross-reactivity and promiscuity between receptor-ligand pairs. Investigations of NKRP1 and Clr binding effects on NK-cell physiology have shown that there are several activating members of the NKRP1s, including Nkrp1a, c, and f, which stimulate NK cell cytotoxic immune responses, and Nkrp1b/d, and Nkrp1g, which inhibit NK cell responses (76, 89, 94, 95). Further, evidence has revealed that Nkrp1f, as well as Nkrp1g, have several interacting partners, with Clr-c, Clr-d, and Clr-g identified as ligands of Nkrp1f and Clr-d, Clr-f, and Clr-g as ligands of Nkrp1g (87, 96) (**Figure 3**). However, the interacting partners of Nkrp1a, Nkrp1c as well as Clr-a and Clr-h are currently unknown.

The mechanisms of signaling through the NKRP1 receptors have been slowly elucidated over time. The activating receptors, appear to follow a canonical FcRγ receptor-mediated ITAM signaling path. For example, Nkrp1c was experimentally demonstrated to require FcRγ binding to promote the secretion of IFNγ in stimulated NK and NKT-cells (97, 98). All of the activating NKRP1 receptors contain residues specifically required for FcRγ association as opposed to DAP12 association as well as the presence of an Lck and PLCγ recruitment motif designated (CxCPR/H) and (YxxL), respectively (99), which is quite similar to the CD4 and CD8 T-cell co-receptor. Nkrp1c was experimentally verified through co-immunoprecipitation to recruit Lck in a fashion similar to the T-cell co-receptor (100). The Nkrp1f receptor, however, while retaining the sequences necessary to recruit the FcRγ receptor appears to be missing the canonical YxxL recruitment motif which may relegate it to a more co-stimulatory role as opposed to directly stimulating (87). In general, FcRγ phosphorylation of activating NKRP1s leads to downstream signaling through the recruitment of Syk and further signal transduction into the cell (101). Conversely, both Nkrp1g and Nkrp1b contain a consensus ITIM motif designated as ΦxYxxΦ (102, 103). In the context of Nkrp1b, this ITIM motif has been shown to recruit SHP-1 in NK cells when stimulated with pervanadate (85) to begin a downstream signaling cascade. Nkrp1g also appears to contain this canonical motif but lacks the recruitment sequence for Lck (87), implying that perhaps it requires a costimulatory molecule or a co-receptor for optimal functioning. This is in contrast to NKR-P1A in humans which contains neither a canonical ITIM nor an Lck recruitment motif but appears to have a non-canonical motif (AxYxxL) that has the theoretical potential to function as a weak ITIM (104). NKR-P1A in NK cells has also been found to co-immuno-precipitate with complexes of Lck, Fyn, and Lyn (105).

## Non-canonical functions of NKRP1 receptors

The function of the NKRP1 receptors is best defined in the context of NK cells since their presence was first detected in NK cells at the time of their initial discovery. While it is now evident that the Nkrp1b receptor mediates inhibitory signaling in NK cells, the ubiquitous expression of its ligand, Clr-b, suggests that this receptor-ligand pair are part of a larger system. In particular, the NKRP1-Clr receptor-ligand interactions are believed to function as an MHC-I-independent recognition system. Clr surface expression signals cell health, protecting cells from NK cell-mediated cytotoxic responses which have been shown to occur when Nkrp1b expressing NK cells engage Clr-b-deficient cells (76, 106, 107). A parallel function has been identified between the human orthologs of this receptor-ligand pair whereby the surface expression of the LLT1 protein is found to be elevated in human prostate cancer, likely a result of a mutation that facilitates tumor cell evasion from immune detection by inhibiting NK cell activity through the Nkrp1a receptor (108). This finding also serves to demonstrate the functional homology of these receptor-ligand interactions as a non-MHC-I method of immune surveillance. This evidence creates the possibility that other functions may be conserved between mice and humans in other aspects of the NKRP1 and Clr family of interacting proteins.

The presence of the NKRP1 and Clr family of receptors and ligands has been documented in other cell types, where they exhibit significantly different functions. For example, the expression of Nkrp1f on DCs and B cells was demonstrated to act as a co-stimulating receptor by engaging Clr-g on activated T-cells prompting enhanced T-cell expansion and IL-2 secretion (109). In addition, Nkrp1g was found to be expressed on CD103^+^ intestinal intraepithelial lymphocytes (110). In this context, the Nkrp1g receptor in tandem with its cognate ligand Clr-f, expressed on neighboring intestinal epithelial cells, was shown to function as a method of immunosurveillance (110). Similarly, Nkrp1b was found to be expressed broadly among gut-associated hematopoietic cells, including gut-resident NK/ILC1 cells, ILC3/LTi cells, ILC2 cells, γδT cells, DC, and macrophage subsets and at higher levels than observed in splenocytes (111). Here, Nkrp1b showed a role in maintaining gut homeostasis, whereby loss of the receptor increased ILCs and γδT cells across different intestinal compartments, but a hyporesponsive capacity among these cell populations that resulted in a breakdown in gut mucosal defense against infection. These observations are believed to reflect a tissue-specific immune detection function of the NKRP1-Clr interactions, which is a result of the NKRP1 receptors being primarily restricted to cells of the hematopoietic compartment while the genetically linked Clr ligands are conversely expressed in a highly variegated fashion throughout the body. While examples of this immune detection function are clearly represented by the NKRP1 receptors with defined ligands, others have been more elusive. Nkrp1c, also known as the NK1.1 antigen, has been the defining receptor for the identification of NK cells for decades. However, the endogenous ligand for Nkrp1c is still unknown. Recent evidence has revealed that Nkrp1c, as well as Nkrp1a and Nkrp1b, interact with the murine cytomegalovirus (MCMV) protein m12. The interaction between Nkrp1b and m12 favors a viral evasion strategy by promoting the inhibition of NK cell cytotoxicity. The m12 binding by the orphan receptors Nkrp1a and Nkrp1c, which have activating receptors, is thought to counterbalance m12 inhibitory functions (112).

## Clr expression and function

As obscure and unexplored as the NKRP1 receptor family is, even less is known about the Clr family of ligands which in some circumstances have unique distribution patterns and signaling functions of their own. For example, Clr-b and Clr-g expression are fairly ubiquitous, being present on most hematopoietic cells (91, 113) and present at varying levels in many epithelial cells with the potential for induced expression under certain conditions such as MCMV infection and kidney reperfusion stress (30, 91, 114). Several Clr-family members also have described roles in contexts other than NK cells. For example, Clr-a and Clr-f exhibit expression within the gut epithelium where they are believed to play a role between immunosurveillance and immune tolerance toward gut microbiota (110, 115). Other Clrs appear to have a more in-depth role in controlling cellular processes and differentiation. For example, Clr-b has been shown to inhibit osteoclast formation *in vitro*, and *in vivo* Clr-b deficient mice appeared to exhibit an aberrant number of osteoclasts resulting in increased bone resorption and lack of bone formation leading to sub-average bone mass in these mice (116). Further evidence of this is provided through genetic studies in human populations that contain an N19K substitution in the homologous LLT1 transcript. This LLT1 mutation was implicated in the increased loss of bone density in postmenopausal women compared to women expressing the normal LLT1 transcript (117). Likewise, the mouse NKRP1:Clr system has been described as a non-MHCI recognition system in mice and it appears to play a similar role in humans, but reports have also described some non-canonical functioning as well. Engagement of the human NKR-P1A receptor on T-cells appears to inhibit T-cell cytotoxicity, in line with the potential of ITIM signaling, but also paradoxically induces proliferation (118).

Similar to the varied roles that NKG2 and Ly49 receptor family members play on different cell types, growing evidence shows the NKRP1 and Clr family of receptor-ligands are also expressed more broadly with unique and varied cell-specific functions. This is exemplified in evidence from an investigation into the function of Clr-f in mice using a mouse knockout model showed a role for Clr-f in kidney health and function. The loss of Clr-f expression resulted in a pronounced kidney-damaging autoimmunity and inflammation, implicating a role for this Clr as an inhibitory ligand for immune cell surveillance in the kidney similar to what was previously posited for Clr-f in the (110, 119). Additionally, Clr-g expressed on lung epithelial cells was identified as a potential new ligand for Nkrp1b, whose interaction with Nkrp1b may represent novel tissue-specific interactions that imprints AMs with their unique metabolic signature and control AM metabolic signaling. This study also revealed new functions for the Nkrp1b receptor on tissue-resident alveolar macrophages (AMs). Resident AMs were found to express Nkrp1b and the loss of this receptor in a knockout mouse model impacted AM survival, immune function, and metabolism. Mice deficient in Nkrp1b were susceptible to *S. pneumoniae* infection due to a gradual collapse of tissue-resident AMs followed by a period of peripheral blood monocyte-mediated renewal. Given that the collapse of the AM population coincided with an accumulation of lipids in AMs and the formation of a foam cell-like phenotype which could be resolved by ex vivo culturing of AMs indicated the importance of Nkrp1b signaling in the lipid processing ability of these cells. Likewise, the observation that Nkrp1b tetramer staining of Clr-g is lost during lung infections with *S. pneumoniae* or influenza A virus infection, points to the role of Nkrp1b-Clr-g interactions as a potential AM immunosurveillance mechanism. This would mirror the MHC-I-independent recognition systems mediated by NK cells through Nkrp1b-Clr-b interactions, or by intraepithelial lymphocytes *via* Nkrp1g-Clr-f interactions (120).

## Concluding remarks

The genetic linkage between the CTLRs and CLECs of the NKC suggests they are part of an ancient recognition system. As this system pertains to NK cell function, the contraction in the number of CTLRs and CLECs within the NKRP1 and Ly49 families in humans compared to mice might be indicative of a replacement of this recognition system with the KIR MHC-I system. Regarding the nature of these families as immune receptors, there are numerous examples in which the interaction between CTLRs and CLECs represents a mechanism of immune surveillance. However, evidence that these interactions also guide other immune adjacent biological processes (e.g., lipid metabolism, cell survival, etc.) should not be ignored. These CTLR functions can be understood as a sensory system for hematopoietic cells to discern the condition of their neighbors and the tissue microenvironments they inhabit *via* CLEC expression profiles. Similarly, the diversity in CTLR homo- or, heterodimeric associations, their binding specificity and ligand promiscuity, and the frequency of atypical signaling motifs among different CTLRs together lead to a broad range of signaling outcomes elicited by CTLRs beyond a straightforward binary activating or inhibitory function. As a result of this cells can interpret a greater complexity of signals in their environment and orchestrate cell responses along a gradient rather than a strict polarization. Although we still have a poor understanding of the CTLR compared to other immune receptors a growing body of evidence is revealing CTLR functions in a greater number of immune processes. CTLR function has expanded from direct recognition of invading pathogens and dead and dying cells to the sensing self-ligands to detect cell transformation and infection, and to the regulation of immune cell responses, cell metabolism, and even recall responses. As investigations of these receptor families continue, the emergence of additional diverse CTLR immune functions is likely.

## Author contributions

MS, BP, SD, and AM wrote and edited the manuscript. All authors contributed to the article and approved the submitted version.
